# The Ratio of IL-6 to IL-4 in Synovial Fluid of Knee or Hip Performances a Noteworthy Diagnostic Value in Prosthetic Joint Infection

**DOI:** 10.3390/jcm11216520

**Published:** 2022-11-03

**Authors:** Xudong Su, Yuelong Chen, Qian Zhan, Bo Zhu, Li Chen, Chen Zhao, Jianye Yang, Li Wei, Zhenghao Xu, Keyu Wei, Wei Huang, Leilei Qin, Ning Hu

**Affiliations:** 1 Department of Orthopedics, The First Affiliated Hospital of Chongqing Medical University, Chongqing 400016, China; 2 Laboratory of Orthopedics, Chongqing Medical University, Chongqing 400016, China; 3 Department of Respiratory and Critical Care Medicine, The First Affiliated Hospital of Chongqing Medical University, Chongqing 400016, China; 4 The Center for Clinical Molecular Medical detection, The First Affiliated Hospital of Chongqing Medical University, Chongqing 400016, China

**Keywords:** prosthetic joint infection, interleukin-6, interleukin-4, diagnosis

## Abstract

The diagnosis of prosthetic joint infection (PJI) is still a challenge, the ratio of interleukin-6 (IL-6) to IL-4 in the joint fluid of knee or hip was used to analyze whether the diagnostic accuracy of PJI can be improved. Between January 2017 and May 2022, 180 patients who developed pain after revision total hip or knee arthroplasty were enrolled retrospectively. 92 patients of PJI and 88 of aseptic failure were included. PJI was as defined by the Musculoskeletal Infection Society (MSIS). The content of IL-6 and IL-4 in synovial fluid of knee or hip were measured, and the areas under the receiver operating characteristic curve (ROC) and IL-6/IL-4 curve were analyzed to obtain a better diagnostic effect. The area under the curve of IL-6/IL-4 in synovial fluid of knee or hip was 0.9623, which was more accurate than ESR 0.5994 and C-reactive protein 0.6720. The optimal threshold of IL-6/IL-4 ratio was 382.10. Its sensitivity and specificity were 81.32% and 98.86%, respectively. The positive predictive value for the diagnosis of PJI was 98.91%. This study showed that the level of IL-6/IL-4 in synovial fluid of knee or hip could further improve the diagnostic accuracy for PJI.

## 1. Introduction

Prosthetic joint infection (PJI) is one of the most common and severe complications after joint arthroplasties [1]. Studies have shown that joint replacement surgeries increase each year, and the incidence of PJI after joint arthroplasties is 1% to 2%, so it will be an even greater problem in the future [2,3]. Therefore, the diagnosis of PJI accurately and efficiently is very important [4,5]. The main difficulty now is how to distinguish PJI and aseptic failure [6]. Early and accurate diagnosis and identification of PJI is critical [4,6,7,8,9,10]. Parvizi et al. proposed a new diagnostic criterion for PJI based on evidence, which is more sensitive than the Musculoskeletal Infection Society (MSIS) criterion [11]. However, no clinical findings or laboratory tests have achieved 100% accuracy under the MSIS criterion, and the definition of PJI remains controversial [12].

Previous studies have found that serum inflammatory biomarkers such as serum C-reactive protein (CRP) and erythrocyte sedimentation rate (ESR), and inflammatory markers such as proinflammatory cytokines interleukin-6 (IL-6) and procalcitonin can play an important role in the diagnosis of PJI [6,8,12,13]. However, due to lack of specificity, these tests are not sufficient to diagnose PJI on their own [14]. Although many markers have been shown to help identify and diagnose PJI and aseptic failure, there is no single detection method that can diagnose PJI [15,16,17]. In view of that, we could diagnose PJI by the combination of clinical manifestations, serum detection and the synovial fluid biomarkers mentioned in the Musculoskeletal Infectious Diseases Society (MSIS) 2013 standard.

IL-6 and IL-4 are pro-inflammatory cytokines and anti-inflammatory cytokines with strong representation, and are mainly produced by activated inflammatory cells (monocytes, microglia, and macrophages) [17,18,19]. Inflammation is an indirect sign of infection [20]. An appropriate inflammation reaction aims to limit inflammation and/or infection to a certain range, remove harmful stimuli and restore microenvironmental dynamics [21]. However, the imbalance caused by the secretion and release of anti-inflammatory and pro-inflammatory factors in PJI will lead to the persistence of inflammation [22]. There are no studies that combine them. Therefore, in this study, we sought to (1) determine whether the ratio of IL-6 and IL-4 in synovial fluid of knee or hip could effectively identify the exact factor (PJI or aseptic failure) in cases where patients undergo revision surgery after total joint arthroplasties failure, and (2) obtain the ratio of the IL-6 and IL-4 in synovial fluid of knee or hip that might ensure the cut-off value in determining PJI.

## 2. Materials and Methods

### 2.1. Methods Study Design, Inclusion, and Exclusion Criteria

From January 2017 to May 2022, we retrospectively analyzed those patients enrolled for revision surgeries due to PJI or implant aseptic failure after knee and hip joint arthroplasties. This study was approved by the institutional review board of the authors’ institution on 26 September 2018 (local ethical committee ref. no: 20187101), and the study was registered in the Chinese Clinical Trial Registry, (registration number: ChiCTR1800020440, approval date 29 December 2018). According to the 2013 Musculoskeletal Infection Society (MSIS) PJI diagnostic criteria, patients were divided into the aseptic failure (including aseptic failure, wear, instability, and misalignment) and the infection groups [15,23]. The guideline includes primary and secondary diagnostic criteria. The latter involves serum C-reactive protein (CRP) level, ESR, synovial white blood cell (WBC) count, neutrophil classification, culture, and leucocyte esterase testing (Table 1) [15]. The “sterile group” was defined as the patients who did not meet the definition of PJI and had not experienced infection or reoperation for at least one year after primary arthroplasty [24]. Exclusion criteria were as follows: (1) inflammatory arthritis, such as rheumatoid arthritis, (2) patients treated with antibiotics within two weeks, (3) ecchymosis, artificial heart valves, or a history of hypercoagulability, (4) other organ infections, such as pneumonia and urinary tract infections, and (5) obese (body mass index [BMI] > 30), heavy smokers or malignant tumors. The following baseline data were recorded: age, sex, body mass index, risk factors for infection, and affected joints. This study conforms to the national ethical standards for biomedical research involving human beings and the latest revised Declaration of Helsinki by the World Medical Association.

### 2.2. Sample Determination

Synovial fluid of knee or hip (1~2 mL) was collected immediately after admission. Samples were centrifuged at 2000 rpm for 10 min within 2 h of collection to remove all cell and particle components. Levels of IL-6 and IL-4 in synovial fluid were determined using the IMMNOLITE 1000 immunoassay system (Siemens Medical, Erlangen, Germany). Blood samples were collected on admission and the day before surgery and then analyzed for serum ESR and C-reactive protein. Synovial fluid was collected for IL-6 and IL-4 analysis and culture before revision arthroplasty, and at least three intraoperative tissue culture samples were taken from patients undergoing revision arthroplasty and cultured for 24 to 48 h (standard culture) and 14 days (extended culture).

### 2.3. Statistical Analysis

Categorical variables were analyzed using SPSS version 25 software (IBM Corp., Armonk, NY, USA). Continuous variables were expressed as mean ± standard deviation, while categorial variables were expressed as counts and percentages. *t*-test was used for continuous variables comparison, and chi-square test was used for categorical variables comparison. The correlation between variables was investigated by the Pearson correlation coefficient. The receiver operating characteristic (ROC) curve and area under curve (AUC) values were analyzed by using MedCalc 15.2.2 software (MedCalc software, Ostend, Belgium). Youden’s J-statistic was used to determine the best cut-off value for the diagnosis of PJI. We also calculated sensitivity, specificity, and accuracy as well as positive and negative predictive values of two serum markers (CRP and ESR) and IL-6/IL-4 in synovial fluid. A value of *p* < 0.05 was considered statistically significant.

## 3. Results

Table 2 shows the demographics of the two groups. These groups included 180 patients; Ninety-two patients (51.11%) were diagnosed with infection (PJI), and 88 patients (48.89%) developed aseptic failure of implants. Baseline characteristics, including age, sex, BMI, and joint type, did not differ significantly between the two groups. As shown in Table 3, there was a statistically significant difference between the infected group and the sterile group of IL-6, IL-4, and IL-6/IL-4 levels (*p* < 0.05). The median values for IL-6, IL-4, and IL-6/IL-4 ratio of the prosthesis infection group were 2024.68, 12.62, 1174.69, respectively, which was significantly higher than for the aseptic failed group (median 300.85, 4.94, 46.65, respectively), with statistical significance (*p* < 0.05). The serum ESR of PJI patients (median 58.00 mm/h) was significantly higher than that of the control group (median 26.10 mm/h) (*p* < 0.0001). Serum CRP level in PJI patients (median, 17.65 mg/L) was also significantly higher than that in aseptic failed patients (median, 5.19 mg/L; *p* < 0.05).

**Figure 1:**
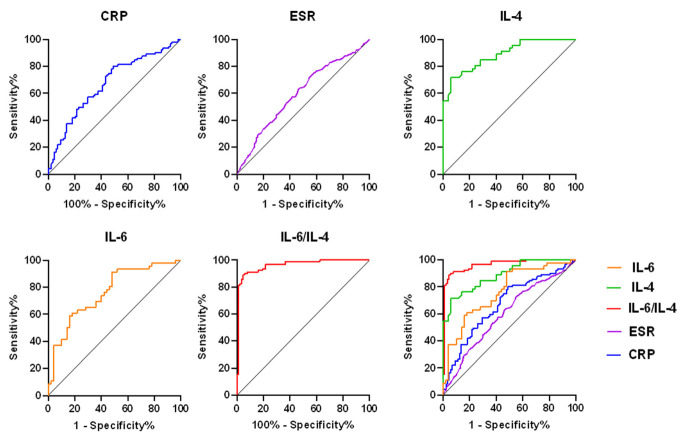
ROC curve shows the PJI predictive value of IL-6 and IL-4 in ratio, IL-6, IL-4, CRP, and ESR. ROC, receiver operating characteristic; PJI, periprosthetic joint infection; CRP, C-reactive protein; ESR, erythrocyte sedimentation rate.

We took the PJI group as positive samples and took the aseptic failed group as negative samples, then modeled receiver operation characteristic based on the IL-6/IL-4 in synovial fluid (Figure 1). Analyzing the ROC, we found that the AUC of IL-6, IL-4, and IL-6/IL-4 in synovial fluid was 0.764 (95% CI, 0.669, 0.859), 0.888 (95% CI, 0.825, 0.951), and 0.9623 (95% CI, 0.9347, 0.9899), respectively. The serum ESR was 0.5994 (95% CI, 0.5285, 0.6704) and serum CRP was 0.6720 (95% CI, 0.5930, 0.7510). Therefore, the AUC of IL-6/IL-4 in synovial fluid was more accurate.

Table 4 shows the standard error and 95% CI of the AUC values. The accuracy values of the synovial fluid for the diagnosis of PJI with a cutoff value of IL-6 (1228.00), IL-4 (7.83), and IL-6/IL-4 (382.10) were 70.83%, 83.33%, and 89.05%, the sensitivities for the diagnosis of PJI were 91.30% (95 CI, 78.31%, 97.18%), 71.74% (95 CI, 56.32%, 83.54%), and 81.32% (95 CI, 71.78%, 88.72%), and the specificities were 52.00% (95% CI, 37.58%, 66.12%), 94.00% (95% CI, 82.46%, 98.44%), and 98.86% (95% CI, 93.83%, 99.97%), respectively. PPV and NPV reached 63.64% and 86.67%, 91.67% and 78.33%, 98.91% and 80.73%, respectively. Table 5 shows the diagnostic characteristics related to inflammatory indicators (CRP and ESR) and IL-6, IL-4, and IL-6/IL-4 in synovial fluid. The positive likelihood ratios for IL-6 IL-4, and IL-6/IL-4 in synovial fluid of patients with PJI were 1.90, 11.96 and 71.33, the negative likelihood ratios were 0.17, 0.30, and 0.19, and the DOR were 11.18, 39.87, and 377.52, respectively. In serum, the positive likelihood ratio, negative likelihood ratios and their DOR for CRP were 1.64, 0.39 and 4.21, while the positive likelihood ratio, negative likelihood ratio and its DOR for ESR were 1.32, 0.62 and 2.13.

## 4. Discussion

The incidence of TJA has increased dramatically in recent years [25,26]. Periprosthetic joint infection (PJI) is one of the most serious complications after artificial joint arthroplasty [1]. The severity of PJI is mainly manifested by high incidence, mortality and high medical expenses [27], which imposes a huge medical and economic burden on society and public health [28]. Because PJI is usually caused by low-virulence microorganisms, it may develop symptoms similar to aseptic failure and different from those typical for acute infection. Therefore, there is a considerable lag in clinical diagnosis of PJI [28,29]. In particular, when PJI presents as a chronic capsular infection, the patient may experience a mild systemic response with normal serum laboratory markers. For this reason, the diagnosis of PJI presents great difficulties [8,30]. Therefore, accurately and timely diagnosis of PJI and accurately distinguishing between PJI and aseptic failure are the key measures to implement effective treatment [31,32]. In this study, the ratio of IL-6/IL-4 in synovial fluid of knee or hip was determined to determine its diagnostic value as a potential screening marker for PJI. The results suggested that the ratio of IL-6/IL-4 in synovial fluid of knee or hip improved the sensitivity and specificity in differentiating PJI from aseptic failure.

Previous studies have identified several humoral anti-inflammatory and pro-inflammatory biomarkers, including IL-1β, IL-1, IL-6, IL-8, granulocyte colony-stimulating factor (G-CSF), tumor necrosis factor α (TNF-α), interferon γ (IFN-γ), α -defensin and β -defensin [18,26,33,34]. It was proved that the level of IL-1 beta and IL-6 in synovial fluid had good sensitivity, specificity and accuracy in the diagnosis of PJI [35]. However, the inflammatory response of PJI is often caused by the imbalance of anti-inflammatory and pro-inflammatory biomarkers [18]. The cytokine IL-6 is a small signaling glycoprotein (molecular weight: 21 KD to 30 KD; 212 amino acids, with variable glycosylation sites), acts as a promoter of inflammation in the inflammatory immune microenvironment, especially in inducing CRP and fibrinogen synthesis in the liver [8,26,36]. The binding of IL-4 to the IL-4 receptor (IL-4R) in inflammation initiates the activation of STAT6 and shifts cells towards an anti-inflammatory phenotype with high ARG-1 CD206 and mannose-receptor (MR), thereby promoting anti-inflammatory cytokines (IL-4, IL-6, IL-10, IL-13, IL-1RA, FIZZ1 and PPARγ) [19,37]. Since serum markers may be affected by acute or chronic inflammation associated with other organs and systems, the diagnosis of PJI may be inaccurate [20,32]. In this study, the ratio of IL-6 to IL-4 in synovial fluid was established as a diagnostic marker of PJI, and the experimental results proved that it had great potential.

The relationship between the IL-6 and the IL-4 in synovial fluid has not been demonstrated in previous reports. In a prospective case-control study, serum IL-6 was shown to be an extremely valuable indicator, as evidenced by its higher accuracy in detecting PJI than that of ESR or CRP [38]. A systematic review conducted in 2018 explored the impact of factors in serum, synovial fluid and tissue on the accuracy of detecting PJI. The results showed that synovial IL-6 had excellent specificity (0.971). The AUC was 0.931 (95% CI 0.858 to 0.973) and its sensitivity was 0.973 [38,39,40]. Previous studies showed that the sensitivity of synovial IL-6 for detecting PJI was 94.59% (95% CI 81.8% to 99.3%), the specificity was 92.86% (95% CI 82.7% to 98.0) and a threshold value of 1855.36 pg/mL. However, the previous studies did not address the interaction of proinflammatory and anti-inflammatory factors [8]. In addition, serum IL-4 levels could also be used to assess early periprosthetic infection, with a specificity of 90.0% and a sensitivity of 60.0% [41]. In this study, the ratio of IL-6 and IL-4 in synovial fluid was used to diagnose PJI for the first time, with ideal sensitivity and specificity, and high accuracy in the diagnosis of PJI.

This study has some limitations. First, we used the 2013 MSIS criteria as the standard for diagnosing PJI [32]. Although the MSIS criteria are widely accepted, they are not the gold standard for the diagnosis of PJI after total joint arthroplasty surgeries. Under the MSIS criterion, we may misclassify patients with or without PJI. Indeed, this may be a challenging situation for all studies evaluating diagnostic tests for PJI infection. Second, this study involved only one study center with a relatively small sample size. However, this preliminary trial showed valuable results and warrants a larger multicenter study to validate the effect of serum and synovial CRP in the diagnosis of PJI. Therefore, a larger prospective multicenter study could be conducted to further validate the trial results. At present, the cost of IL-4 and IL-6 analysis in research centers is low, and it is relatively common. Therefore, it is feasible to use IL-4 and IL-6 for research purposes and in daily practice. Finally, to eliminate confounding factors, patients who had recently used antibiotics were excluded from this study. However, this may limit the generalizability of this study.

## 5. Conclusions

In this study, the levels of IL-6/IL-4 in synovial fluid of knee or hip of PJI patients were significantly increased. Based on MSIS criteria, the IL-6/IL-4 value reflected a high diagnostic sensitivity and specificity. The ratio of IL-6 to IL-4 in synovial fluid is a promising synovial fluid biomarker. This synovial fluid biomarker can not only improve the accuracy of PJI diagnosis, but also provide continuous guidance for antimicrobial treatment and monitor the effectiveness of surgical treatment; it may be included in the diagnostic criteria in the future.

## Figures and Tables

**Table 1:** The Musculoskeletal Society 2013 Definition of PJI, periprosthetic joint infection. MSIS, Musculoskeletal Infection Society. ^a^ One of the 3 criteria (1, 2, or 3) must be met for diagnosis of PJI.

Musculoskeletal Infectious Diseases Society MISIS Definition of Prosthetic Joint Infection PJI ^a^	
1	There is a sinus tract communicating with the prosthesis; or
2	Two positive periprosthetic cultures with phenotypically identical organisms; or
3	When 3 of the following 5 criteria exist:
	(a) serum C-reactive protein AND erythrocyte sedimentation rate
	(b) Elevated synovial fluid white blood cell count OR++ change on leukocyte esterase test strip
	(c) Elevated synovial fluid polymorphonuclear neutrophil percentage
	(d) Positive histological analysis of periprosthetic tissue
	(e) A single positive culture

**Table 2:** Demographic data for the study population.

Characteristic	Aseptic (N = 88)	Infected (N = 92)	*p*-Value
Age (y)	65.61 ± 13.25	66.75 ± 10.89	0.532
Body Mass Index BMI (kg/m^2^)	23.67 ± 4.06	23.75 ± 3.18	0.873
Gender			
Male	36 (20%)	43 (23.89%)	0.434
Female	52 (28.89%)	49 (27.22%)	
Joint type			
Knee	33 (18.33%)	45 (25.00%)	0.1238
Hip	55 (30.56%)	47 (26.11%)	

**Table 3:** Analysis of inflammatory markers in patients with infected and aseptic revision arthroplasty CRP, C-reactive protein; ESR, erythrocyte sedimentation rate.

Inflammatory Marker		Hip + Knee		
		Aseptic (N = 88)	Infected (N = 91)	*p*-Value
ESR (mm/h)	Median	26.10	58.00	<0.0001
	P25	15.75	30.00	
	P75	50.25	84.50	
CRP (mg/L)	Median	5.19	17.65	0.0017
	P25	3.18	7.15	
	P75	17.85	43.98	
IL-6/IL-4	Median	46.65	1174.69	0.0335
	P25	20.80	347.37	
	P75	111.16	2692.32	
IL-6	Median	300.85	2024.68	<0.0001
	P25	92.29	710.56	
	P75	1325.15	3402.04	
IL-4	Median	4.94	12.62	<0.0001
	P25	1.78	9.34	
	P75	8.39	15.56	

**Table 4:** Sensitivity, Specificity, PPV, NPV, and Accuracy of Inflammatory Markers. PPV, positive predictive value; NPV, negative predictive value; ESR, erythrocyte sedimentation rate; CRP, C-reactive protein; AUC, area under the curve; CI, confidence interval.

Parameters	ESR (mm/h)	CRP (mg/L)	IL-6/IL-4	IL-6	IL-4
AUC (95% CI)	0.5994 (0.5285, 0.6704)	0.6720 (0.5930, 0.7510)	0.9623 (0.9347, 0.9899)	0.764 (0.669, 0.859)	0.888 (0.825, 0.951)
Cutoff level	28.50	6.37	382.10	1228.00	7.83
Sensitivity (95% CI)	71.77% (62.99%, 79.49%)	80.22% (70.55%, 87.84%)	81.32% (71.78%, 88.72%)	91.30% (78.31%, 97.18%)	71.74% (56.32%, 83.54%)
Specificity (95% CI)	45.45% (36.38%, 54.76%)	51.14% (40.25%, 61.95%)	98.86% (93.83%, 99.97%)	52.00% (37.58%, 66.12%)	94.00% (82.46%, 98.44%)
PPV (%)	46.19	51.70	98.91	63.64	91.67
NPV (%)	70.97	79.28	80.73	86.67	78.33
Accuracy (%)	55.76	91.31	89.05	70.83	83.33

**Table 5:** Performance of the ESR, CRP, IL-6, IL-4, and IL-6/IL-4 for diagnosing PJI. CRP, C-reactive protein; ESR, erythrocyte sedimentation rate; PJI, periprosthetic joint infection; PPV, positive predictive value; NPV, negative predictive value; LR, likelihood ratio; DOR, diagnostic odds ratio.

Test	ESR (mm/h)	CRP (mg/L)	IL-6	IL-4	IL-6-/IL-4
Sensitivity (%)	71.77	80.22	91.30	71.74	81.32
Specificity (%)	45.45	51.14	52.00	94.00	98.86
PPV (%)	46.19	51.70	63.64	91.67	98.91
NPV (%)	70.97	79.28	86.67	78.33	80.73
LR+	1.32	1.64	1.90	11.96	71.33
LR−	0.62	0.39	0.17	0.30	0.19
DOR	2.13	4.21	11.18	39.87	377.52

## Data Availability

The data that support the fundings of this study are available from the corresponding author, Ning Hu, upon reasonable request.

## Abbreviations

PJI: Prosthetic joint infection; IL-6: Interleukin-6; IL-4: Interleukin-4; ROC: Receiver operating characteristic; CRP: C-reactive protein; ESR: Erythrocyte Sedimentation Rate; SF: Synovial fluid; CI: Confidence interval; PPV: Positive predictive value; NPV: Negative predictive value; AUC: Area under the curve; MSIS: Musculoskeletal Infection Society; MR: mannose-receptor; G-CSF: Granulocyte colony-stimulating; TNF-α: Tumor necrosis factor α; IFN-γ: Interferon γ.
